# In-Plane Radiation of Surface Plasmon Polaritons Excited by Free Electrons

**DOI:** 10.3390/mi15060723

**Published:** 2024-05-30

**Authors:** Ping Zhang, Yin Dong, Xubo Li, Xinxin Cao, Youfeng Yang, Guohao Yu, Shengpeng Yang, Shaomeng Wang, Yubin Gong

**Affiliations:** 1 School of Electronic Science and Engineering, University of Electronic Science and Technology of China, Chengdu 610054, China; 2 Suzhou Institute of Nano-Tech and Nano-Bionics (SINANO), CAS, Suzhou 215123, China; 3 Yangtze Delta Region Institute (Quzhou), University of Electronic Science and Technology of China, Quzhou 324003, China

**Keywords:** surface plasmon polaritons, free electron excitation, lateral radiation

## Abstract

Surface plasmon polaritons (SPPs) have become a research hotspot due to their high intensity and subwavelength localization. Through free-electron excitation, a portion of the momentum of moving electrons can be converted into SPPs. Converting highly localized SPPs into a radiated field is an approach with the potential to aid in the development of a light radiation source. Reducing losses of SPPs is currently a critical challenge that needs to be addressed. The lifetime of SPPs in metal films is longer than that in metal blocks. Traditional optical gratings can transform SPPs into radiation to avoid the decay of SPPs in metal; however, they are created by etching metal films, so they tend to alter the dispersion characteristics of these films and will emit radiation in the direction perpendicular to the metal surface. This paper proposes an approach to converting the SPPs of a metal film excited by free electrons into a radiation field via lateral grating and obtaining in-plane radiation. We investigate the properties of SPP lateral radiation. The study of lateral radiation from metal films holds significant importance for SPP radiation sources and SPP on-chip circuit development.

## 1. Introduction

An effective way of generating and amplifying electromagnetic waves is to transfer the momentum of moving electrons to electromagnetic waves. This traditional method requires synchronization between the velocity of electrons and electromagnetic waves [1,2], so periodical structures are commonly used to obtain such synchronization [3,4,5].

Surface plasmon polaritons (SPPs) are naturally slow waves [6,7,8,9] and cannot be excited simply by casting light onto their surfaces directly because the wave vectors are not matched. Some structures are used to compensate for the wave vector, such as grating, the Otto configuration, or the Kretschmann–Raether configuration. The wave vector of moving electrons is exactly synchronized with SPPs; that is, the velocity of moving electron is synchronized with the phase velocity of SPPs. Therefore, a moving electron can be used to excite SPPs directly.

Lecante et al. used electrons to describe parabolic trajectories; the electrons were deflected off the metal surface via a bias potential and excited the surface plasmons [10]. Subsequent studies managed to aim a STEM aloof beam parallel to the planar surfaces of MgO cubes. The aloof configuration has recently been reconsidered and compared with near-field optical microscopy [10,11,12,13]. Until now, this excitation method has been used for the development of light sources, terahertz sources, and even X-ray sources [14,15,16,17,18,19].

The phase velocities of SPPs excited by electrons are synchronized with the velocity of a moving electron. SPPs excited by electrons are localized on the metal surface and generally radiate through the periodic structure. The commonly used structure in this regard is a metal surface etching grating, but at the same time, the change in surface morphology will bring strong dispersion change, which will affect the characteristics of SPPs [20,21]. If the smooth surface is retained, a periodic dielectric medium can be loaded below [22,23,24,25], but processing becomes cumbersome. In this structure, the radiations are projected along the vertical plane.

In addition to the transformation of SPPs into Smith–Purcell radiation (SPR) [21,22,23,24,25,26,27], attempts have also been made to convert SPPs into Cherenkov radiation [28,29,30,31,32] and even generate Cherenkov radiation without a threshold requirement for the electron velocity in a hyperbolic metamaterial [29]. As opposed to transformation to Cherenkov radiation, the most important feature of the transformation to SPR via periodic structures is the absence of a threshold requirement for electron velocity. As a supplement to conventional gratings, various metasurfaces or metamaterials are increasingly being applied to the study of the conversion of localized fields into radiation [33,34]. In 2015, Tiejun Cui et al. proposed a method in which a link is established between SPP waves and radiation waves in a highly controllable way using ultrathin corrugated metallic strips, opening up an avenue for designing new kinds of microwave and optical elements in engineering [35]. Furthermore, achieving plane waves along different angles by controlling phase gradients has also been studied [36]. In 2020, Mengxuan Wang, et al. realized vortex Smith–Purcell radiation generation [37]. In 2023, L. Wang et al. realized giant and broadband THz and IR emission [38].

**Figure 1:**
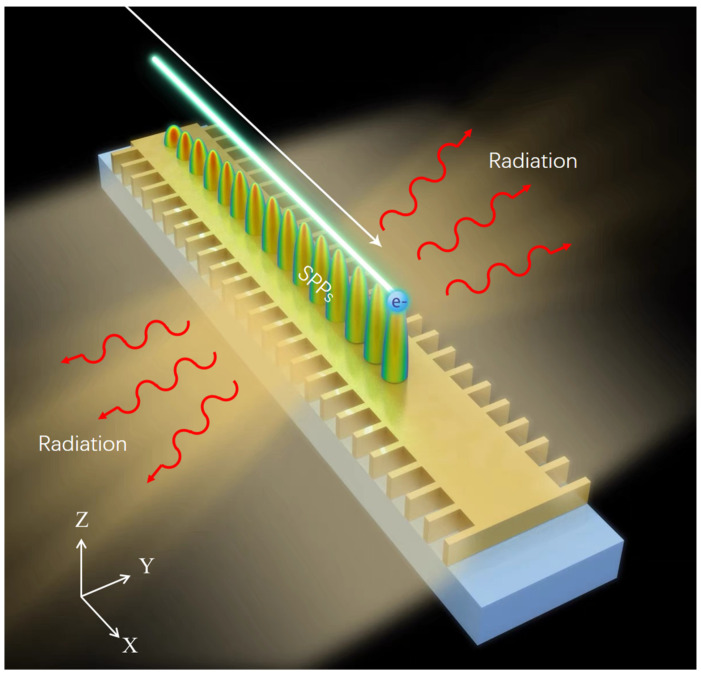
Schematic diagram illustrating the in-plane radiation of SPPs. The SPPs’ lateral radiation structure was obtained by etching the grating on the edge of the metal film. When an electron moves above the metal film, the SPPs are excited and then transform into radiation via the lateral grating.

Most of the previous discussions on transforming SPPs into either SPR or Cherenkov radiation have focused on the radiation properties of the radiation in the perpendicular plane (the plane perpendicular to the metal surface). In this article, based on the localization and the lateral expansibility of SPPs excited by electrons on metal film [39], it was determined that the proposed lateral radiation structure, shown in Figure 1, could not only produce in-plane radiation but also preserve the original dispersion characteristics of the metal film to a great extent, which is important for the later application of SPPs in plane circuits.

**Figure 2:**
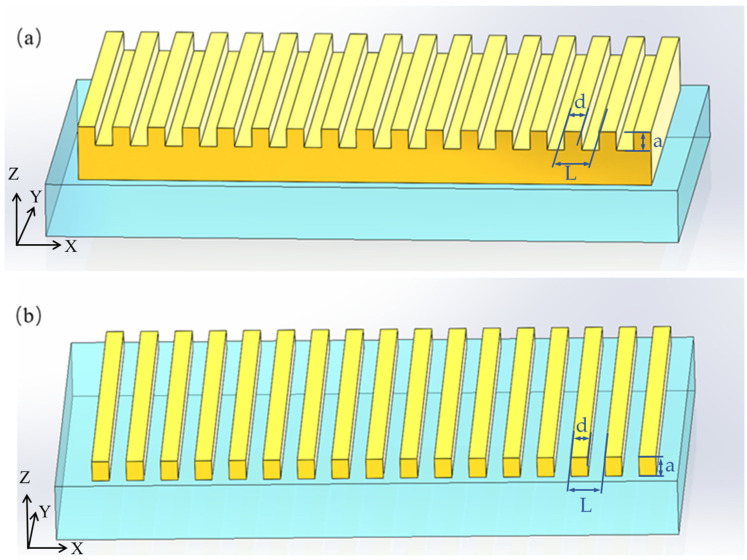
Two conventional diagrams for SPP radiation excited by parallel-moving electrons in the x direction. (**a**) For a metal block, a grating can be etched on the surface. (**b**) For a metal film, the film is etched into strips. For the structure (**a**,**b**), the radiation direction is mainly concentrated in the plane perpendicular to the metal surface (x-z plane).

Figure 2 shows two traditional periodic structures, which are used to transform SPPs into vertical plane radiation. Figure 2a shows the etched grating structure on the metal film. The grating period L is 100 nm, the tooth width d is 50 nm, and the grating depth is 50 nm. In general, radiation efficiency will increase with an increase in grating depth. However, this structure greatly affects the dispersion relations and also excites surface waves, resulting in a near field that is a combination of SPPs and surface waves. The SPPs propagating along a corrugated surface decay into light of high intensity on a non-smooth surface. The measurement of this light’s intensity and angular distribution allows the determination of the roughness parameter, r.m.s. height, and correlation length. To increase radiation intensity, metal stripe structures have been investigated, as shown in Figure 2b.

Here, we use a periodical boundary loading method that involves etching patterns onto the side of the metal film, taking advantage of the longer lifetimes of SPPs in films [40], to convert SPPs into radiation fields. This method may have significant potential for converting SPPs supported by two-dimensional electron gas. We used the lateral radiation of SPPs in a metal film as an example to investigate the physical mechanism of this method and the rules of the generation and conversion of SPPs.

## 2. SPPs in a Metal Film Excited by Free Electrons

Compared with the metal grating structure, the properties of SPPs in a smooth metal film offer two advantages. 1. The SPPs are easier to characterize and control because this method avoids the influence of the periodic structure on dispersion, so it can better reflect the characteristics of the metal film. 2. The SPPs in the metal film have a long lifetime.

**Figure 3:**
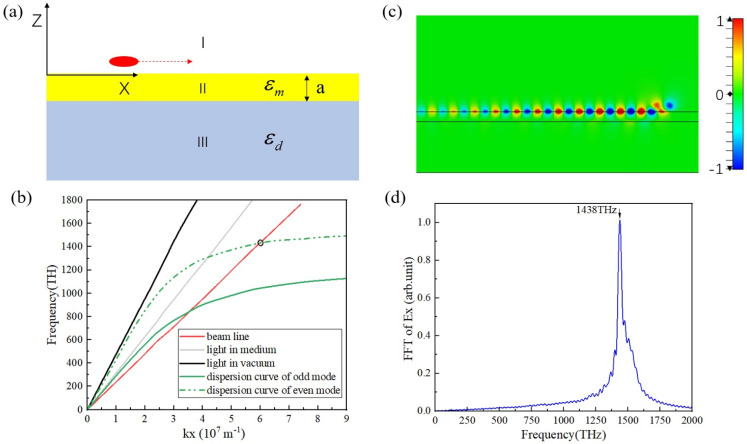
(**a**) Schematic diagram of SPPs in metal thin-film structure excited by moving electrons. (**b**) Dispersion diagram, where the green curve is the SPPs’ dispersion curve; the black line is the light line in a vacuum; the gray line is the light line in the medium; and the red line is the electron line at β = 0.5 (β is the ratio of electron speed to light speed). (**c**) A contour map of Ez. (**d**) The spectrum of Ez.

For metal films on a dielectric medium structure, as shown in Figure 3a, the dispersion equation is [19]

(1) $$\frac{\left(\varepsilon_{m}k_{z1}-k_{z2}\right)}{\left(j\varepsilon_{m}k_{z1}-\varepsilon_{d}k_{z2}\right)}e^{-k_{z2}a}=\frac{\left(\varepsilon_{m}k_{z1}+k_{z2}\right)}{\left(j\varepsilon_{m}k_{z3}+\varepsilon_{d}k_{z2}\right)}e^{k_{z2}a}$$

where $k_{zn}=\sqrt{\varepsilon_{n}k_{0}^{2}-k_{x}^{2}}$, $k_{0}=\omega /c$, $k_{x}$ denote a longitudinal wave vector; a is the metal film’s thickness; and $\varepsilon_{d}$ is the relative permittivity of the substrate. SiO_2_, with a relative permittivity equal to 2.3, was chosen as the substrate. In order to avoid the Cerenkov condition being satisfied due to the $\varepsilon_{\infty }$ in the modified Drude model, we chose the simplest Drude model instead of the modified Drude model, featuring the following parameters [41]:

(2) $$\frac{\varepsilon_{m}\left(\omega \right)}{\varepsilon_{0}}=1-\frac{\omega_{p}^{2}}{\omega^{2}-\gamma^{2}}+i\frac{\omega_{p}^{2}\gamma }{\omega (\gamma^{2}+\omega^{2})}$$

where $\omega_{p}=1.37\times 10^{16}\;s^{-1}$, and $\gamma =3.68\times 10^{13}\;s^{-1}$. By calculating Equation (1), we can obtain the dispersion curve, as shown in Figure 3b. The frequency of the SPPs is determined by the intersection of the electron beam line and the dispersion curve, which means that the electron velocity and the phase velocity of the SPPs meet the synchronization condition at this point. For a metal film, there are two points of intersection, and the higher-frequency point of intersection mainly describes SPPs at the interface between the air and metal.

The SPPs excited by moving free electrons were studied using the Particle-In-Cell (PIC) simulation method [42]. We used the particle studio of CST to perform the simulation. In the simulation, we used a longitudinally Gaussian-distributed electron bunch with a radius of 15 nm as the excitation source, with a charge quantity of 1.0 × 10^−17^ C, a longitudinal size of 60 nm, and an initial electron velocity of 0.5 c. As shown in Figure 3a, we added a metal film with a thickness of 50 nm to the dielectric substrate, where the dielectric substrate material is SiO_2_ and the relative permittivity is 2.3. We adopted the Drude model to describe the metal film in the CST, and its specific parameters are shown in Equation (2). In the later in-plane radiation study, we placed the whole model in an air box with the boundary condition, which was set to be the perfectly matched layer (PML), to simulate an infinite space so that we could obtain the radiation characteristics of the SPPs in the horizontal and vertical directions, respectively.

The contour map of Ez in z-x plane and the time domain waveform of SPPs can be obtained, as shown in Figure 3c,d. According to the simulation results, only the odd mode exists at the metal–air interface, and its intensity is significantly greater than that of the even mode because the excitation efficiency of the odd mode is much higher than that of the even mode for the electrons moving above the metal film. Therefore, our subsequent investigation only focuses on the odd mode of SPPs. The wavelength of SPPs is about 104 nm, as shown in Figure 3c, and this agrees with the calculation, that is, $\lambda_{\mathrm{SPPs}}=2\pi /k$, based on Figure 3b.

**Figure 4:**
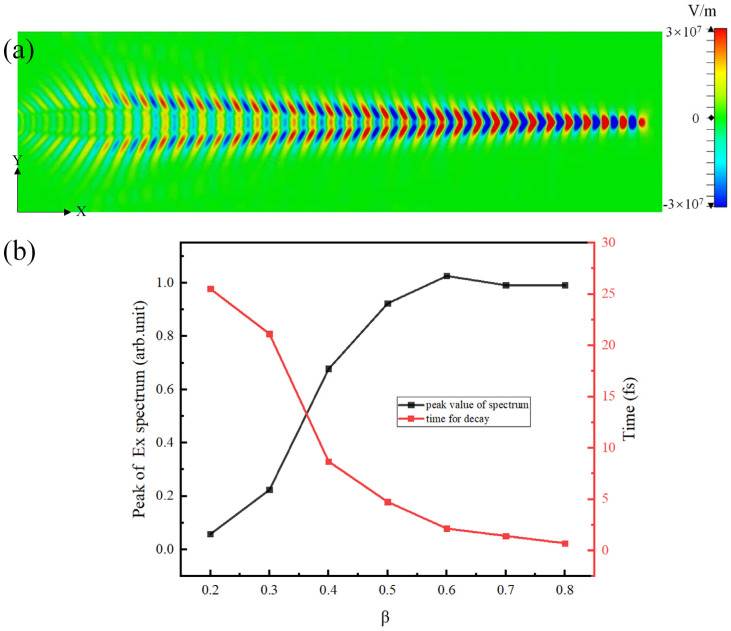
(**a**) A contour map of an electric field in the x–y plane when t is 25 fs. (**b**) The intensity and the decay time of SPPs vs. electron velocity.

Figure 4a shows the electric field distribution in the x–y plane. It should be noted that the SPPs in the metal not only propagate in the x direction but in the y direction as well, and they are amenable to Cherenkov radiation in this plane. The propagation form of SPPs on the metal surface creates conditions for lateral radiation. In order to make the radiation stronger, we discuss the influence of electron velocity on the excitation intensity and lifetime of SPPs.

The relations between SPPs intensity and electron velocity are discussed as follows. It was found that intensity increases with an increase in β but tends to gradually saturate after reaching the point where β > 0.5, as shown by the black line in Figure 4b. The red line in Figure 4b shows the decay time of SPPs. It shows that although the intensity is low when β is small, the attenuation is slow. We made a trade-off between the intensity and attenuation time of SPPs and set β equal to 0.5.

**Figure 5:**
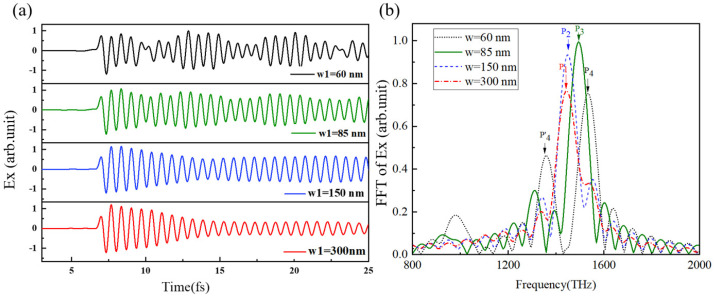
Observation points were set on the metal surface below the electron with different film widths to obtain (**a**) SPPs’ time domain waveform and (**b**) corresponding spectrum.

The simulation results in Figure 5a show that the reflected wave occurs at the metal’s edge when the metal’s transverse width w_1_ is small. The reflected wave will engage in coupling with the propagating SPPs. We set w_1_ to 60 nm, 85 nm, 150 nm, and 300 nm and investigated its influence on the propagation and decay of SPPs. The results are shown in Figure 5b. As w_1_ gradually decreases, the frequency of the SPPs adopts a slight blue shift, from p1 to p4. When the transverse width is 150 nm, the time domain signal of metal SPPs gradually increases and becomes stable after slight attenuation. When w_1_ is smaller than the wavelength of SPPs (104 nm), the reflected wave of SPPs at the edge will be coherent with the SPPs propagating forward, resulting in an obvious resonance effect.

It can be clearly seen from the time domain waveforms that the attenuation of SPPs increases with a wider metal film, because the intensity of the reflected wave decreases. When the width is relatively low, there is an obvious spectrum split due to resonance. When w_1_ = 60 nm, there are two peak values, p4 and p4′. Therefore, in the subsequent research model, we set the transverse width of the metal film to 100 nm to avoid an obvious spectrum split.

**Figure 6:**
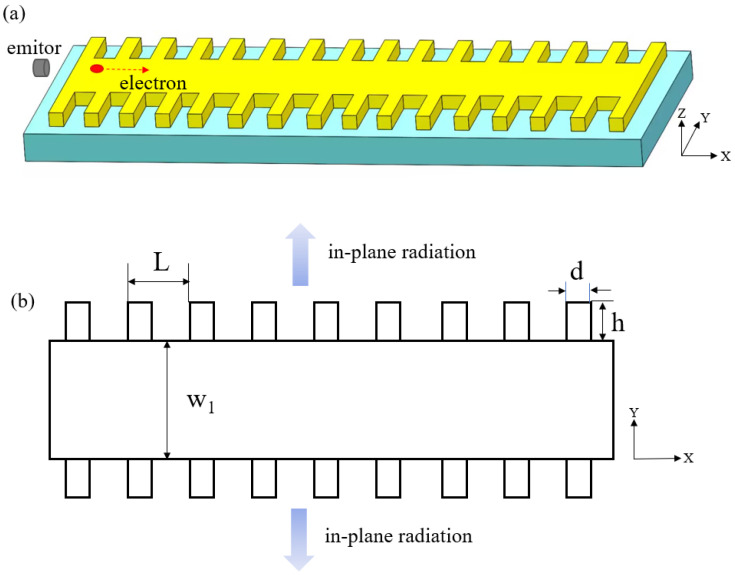
The SPPs’ lateral radiation structure, with electrons moving from left to right. (**a**) Three-dimensional diagram of the structure. (**b**) Radiation direction diagram and structural parameters.

Although SPPs supported by a metal film can propagate over longer distances, the fabrication of a grating to emit radiation on the metal film is much harder. In addition, even though the thickness is suitable for etching a grating, the periodical structure on the film will greatly affect the dispersion of SPPs. Based on the transverse expansion phenomenon occurring as SPPs propagate, which can be observed in Figure 4a, we propose a structure wherein the grating is etched into the edge of the metal film. This structure allows us to preserve the desirable properties of SPPs on a smooth film surface while achieving lateral radiation, as shown in Figure 6. The structural parameters are shown in Figure 6b.

## 3. In-Plane SPPs’ Lateral Radiation

A series of periodical rectangular teeth were fabricated on the edge of the metal film to realize lateral radiation. When electrons move above the metal film, SPPs are excited and expand transversely, as shown in Figure 4a. Radiation is generated when the SPPs reach the periodical boundary.

The radiation frequency is determined by the frequency of SPPs; however, the radiation angle can still be predicted according to the Smith–Purcell radiation principle, and the corresponding formula is shown as below

(3) $$\cos\theta =\frac{1}{\beta }-\frac{L}{\lambda }$$

where *λ* is the wavelength of the radiation wave, *L* is the period of the grating, *β* is the ratio of electron speed to light speed c, and *θ* is the radiation angle of the radiation wave.

Firstly, we changed the periodical length L in order to investigate its influence on lateral radiation. The radiation consists largely of SPP radiation, so its frequency corresponds to that of SPPs. When the radiation angle *θ* equals 90°, we can use Equation (3) to calculate the period of a tooth, which approximately equals the wavelength of SPPs, namely, 104 nm. In the simulation model, the period number, the metal film’s thickness, and electron velocity were set to 40, 50 nm, and 0.5 c, respectively.

**Figure 7:**
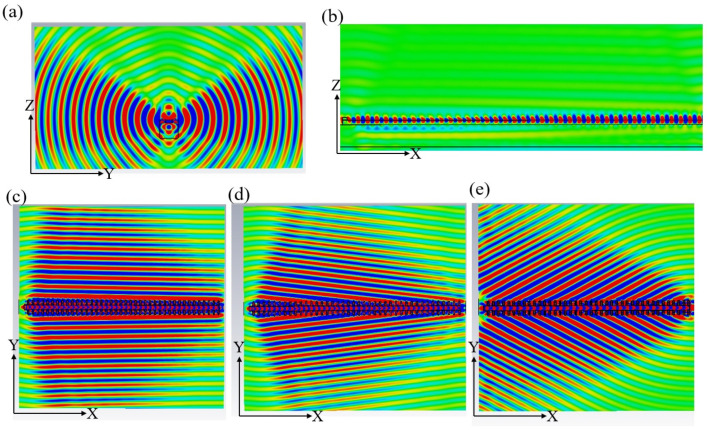
Lateral radiation phenomenon. The contour map of Ex for different periods *L*. (**a**) Radiation diagram in the plane (y-z plane) perpendicular to the electron motion direction at a period of 104 nm; (**b**) radiation diagram in the plane (x-z plane) at a period of 104 nm; (**c**) radiation diagram in the plane (x-y plane) at a period of 104 nm; (**d**) radiation diagram in the lateral plane (x-y plane) at a period of 80 nm; (**e**) radiation diagram in the lateral plane (x-y plane) at a period of 110 nm.

Here, we set the period *L* to 80 nm, 104 nm, and 110 nm, respectively, and investigate the lateral radiation of SPPs. The tooth width d and depth h are 50 nm and 100 nm. The results are shown in Figure 7. When *L* is 104 nm, the contour maps of Ex in the y-z plane, x-z plane, and x-y plane are shown in Figure 7a, 7b, and 7c, respectively. Figure 7a shows that the type of radiation is lateral radiation, and Figure 7c shows that the radiation angle is 90°. When *L* is 80 nm, the radiation angle is less than 90°, as shown in Figure 7d, while when *L* is 110 nm, the radiation angle is more than 90°, as shown in Figure 7e.

**Figure 8:**
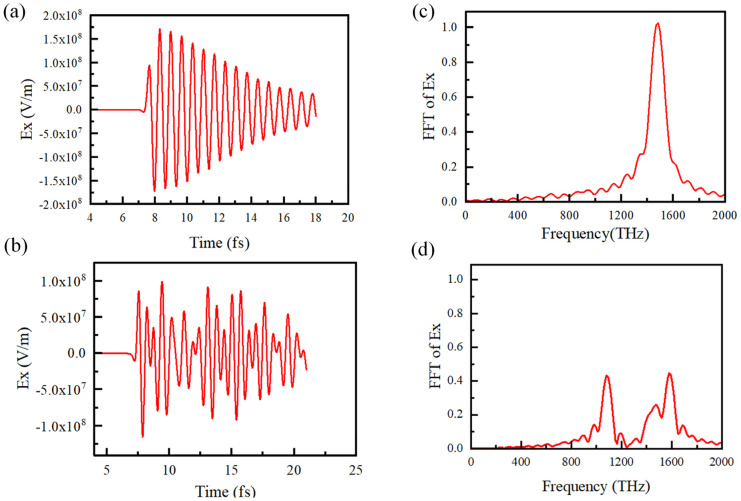
Electrons moving above the lateral radiation structure and the traditional grating structure. (**a**) The time–domain waveforms for the lateral radiation structure and (**c**) its spectrum. (**b**) The time–domain waveforms for the grating structure and (**d**) its spectrum.

In the subsequent simulation, we investigated the effects of SPPs and radiation fields on lateral radiation structure and the metal grating structure. For the purposes of comparison, the grating depth h and period *L* are 50 nm and 104 nm, respectively. The same electron beam was used for excitation. The SPPs of these two structures are shown in Figure 8. Figure 8a shows the SPPs of the metal film in the lateral radiation structure excited by electrons, which has a relatively pure frequency. Figure 8b shows the circumstance wherein the metal grating is etched on the metal block. Due to the introduction of the grating onto the metal film, the field strength on the surface of the metal grating structure decreases, and the dispersion properties of SPPs cannot be maintained independently, so two frequencies are generated. Upon comparing Figure 8c,d, it can be seen that the SPPs of the metal film have a purer frequency and a greater single-frequency intensity.

**Figure 9:**
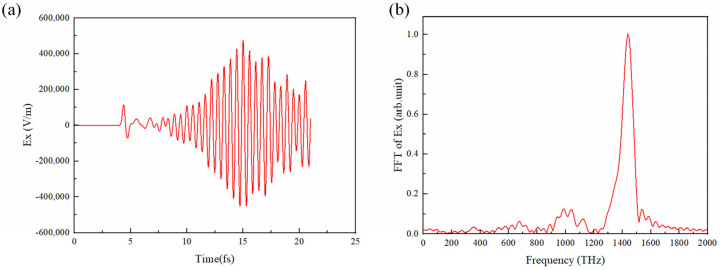
The lateral radiation fields. (**a**) The time–domain waveform of radiation field Ex; (**b**) the corresponding spectrum of the radiation field.

Lateral radiation is emitted in two directions in x-y plane, as shown in Figure 7b. We measured the radiation field in one of the directions in the far-field region (the result is shown in Figure 9a) and performed Fourier analysis on it, as shown in Figure 9b. Its spectrum shows that it is exactly the same radiation converted from the SPPs; the spectrum peak agrees with that in Figure 8c.

## 4. Discussion

**Figure 10:**
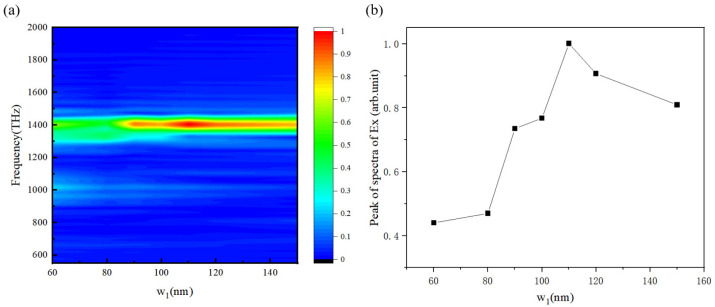
(**a**) Radiation spectra under different film widths. (**b**) Relationship between film width and peak intensity of radiation spectra.

For the lateral radiation structure, decreasing film width w_1_ will bring about the resonant effect. We studied the radiation spectrum for different film widths, and the results are shown in Figure 10a. They show that when the film width is about equal to the wavelength of SPPs, the level of radiation reaches the maximum value. When the film width decreases, the resonant effect brings about an energy split, and they are both transformed into radiation. The relationship between film width and peak intensity of the radiation spectra is shown in Figure 10b.

Like traditional grating radiation, the tooth parameters will greatly affect the radiation effect. But there are also essential differences. The tooth profile here will not affect the radiation frequency because the radiation energy is dominated by the SPPs of the metal film. Here, we optimize the tooth profile and study the effects of the period, grating depth h, and grating teeth width d on the radiation intensity in detail.

The influence of tooth period is shown in Figure 7. Because this constitutes the radiation of SPPs, the period will hardly affect the frequency of SPPs, and the period will only determine the angle of SPPs. This is also a prominent feature of lateral radiation.

**Figure 11:**
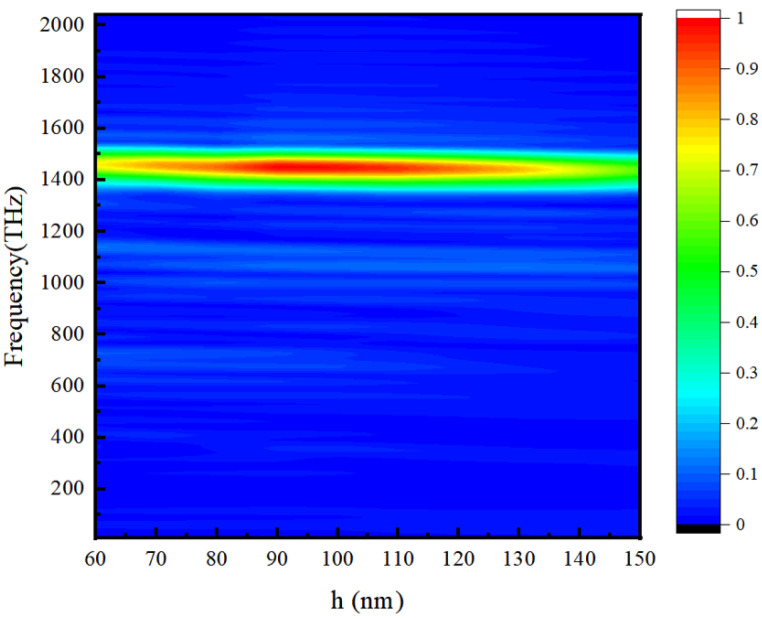
Radiation spectrum corresponding to the depths of different teeth.

Now, we study the grating depth h and record the radiation spectrum corresponding to different grating depths, as shown in Figure 11. It can be seen from the results that, firstly, when we change the grating depth, the radiation always adopts a certain frequency, which is the confirmation of SPP conversion, and it is different for SPP radiation in the grating structure, in which the grating depth will affect the dispersion of SPPs. Secondly, as the grating depth increases, the radiation intensity first increases and then decreases, and when the depth is about equal to the wavelength of SPPs, the radiation intensity is the strongest.

**Figure 12:**
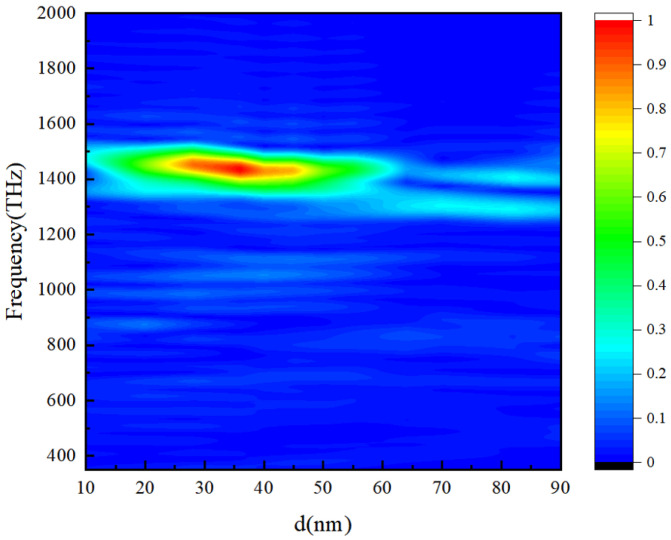
Radiation spectrum corresponding to different duty cycles of teeth (1-d/P).

Next, we study the relationship between the duty cycle of the grating teeth and radiation intensity, as shown in Figure 12. When the width of the teeth is between 30 and 40 nm, the radiation is strongest; however, the radiation frequency is almost the same. In a traditional grating, the radiation frequency is also affected by the grating duty cycle, including with respect to two points: 1. the duty cycle induces surface waves, and 2. the duty cycle affects the intensity of SPPs excited by electrons. In our structure, SPPs’ characteristics are determined by the smooth metal film, and the duty cycle of the teeth (1-d/P) just determines the efficiency of SPPs converting into a radiation field.

## 5. Conclusions

The attenuation of SPPs supported by a metal film is lower than that of SPPs supported by a metal block. We propose a structure fabricated by etching a grating onto the side of the metal film. This structure can not only convert the SPPs into radiation fields but also preserve the desired characteristics of SPPs on the metal film. The corresponding results were verified via theoretical analysis and particle-in-cell simulation. The proposed lateral radiation method also provides an idea for the future study of two-dimensional electron gas SPPs radiation sources, and it will also be used to develop on-chip SPP circuits.

## Data Availability

The original contributions presented in the study are included in the article, further inquiries can be directed to the corresponding authors.
